# Isolated Ascites on CT After Blunt Trauma: A Sign of Intraperitoneal Bladder Rupture

**DOI:** 10.7759/cureus.20479

**Published:** 2021-12-17

**Authors:** Xiaoni Zhao, Pierre D Maldjian

**Affiliations:** 1 Department of Radiology, Rutgers New Jersey Medical School, Newark, USA

**Keywords:** ct cystography, abdominal ct, isolated free fluid, blunt abdominal trauma, bladder rupture

## Abstract

We report a case of intraperitoneal bladder rupture in a 24-year-old man who was struck by a motorcycle. Initial contrast-enhanced CT scan shortly after presentation to our emergency department demonstrated simple free fluid within the upper abdomen and pelvis. Delayed CT scan of the pelvis showed contrast extravasation into the perineal cavity. CT cystography showed rupture of the bladder dome with active contrast extravasation. This case illustrates that intraperitoneal bladder rupture should be considered as an etiology for otherwise unexplained ascites after blunt abdominal trauma. Delayed CT and CT cystography should be considered for further evaluation.

## Introduction

Bladder injury is a rare consequence of blunt abdominal trauma [1]. CT is usually the first imaging study performed to screen for abdominal organ injures. It is important for radiologists to realize that isolated ascites on CT could be a sign of intraperitoneal bladder rupture. We present an unusual case of unexplained ascites in such a patient where delayed CT was crucial for confirming the diagnosis of bladder injury.

## Case presentation

**Figure 1:**
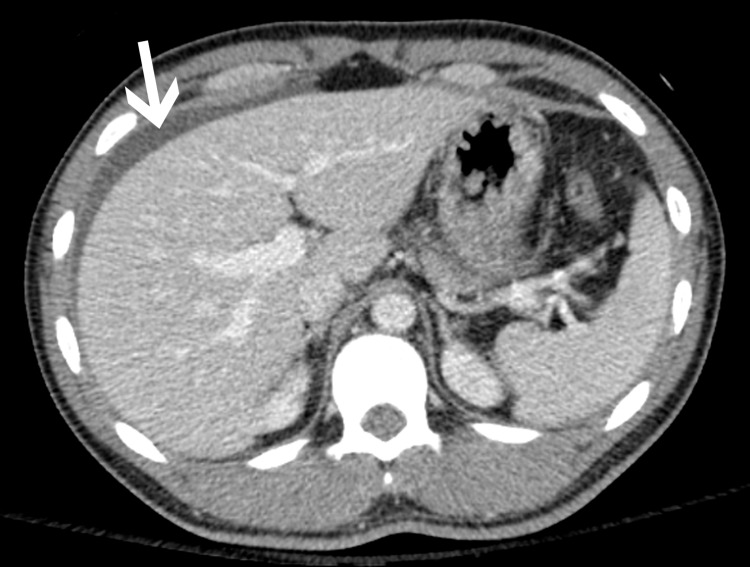
Axial CT image through the upper abdomen shows ascites (white arrow) adjacent to the liver.

**Figure 2:**
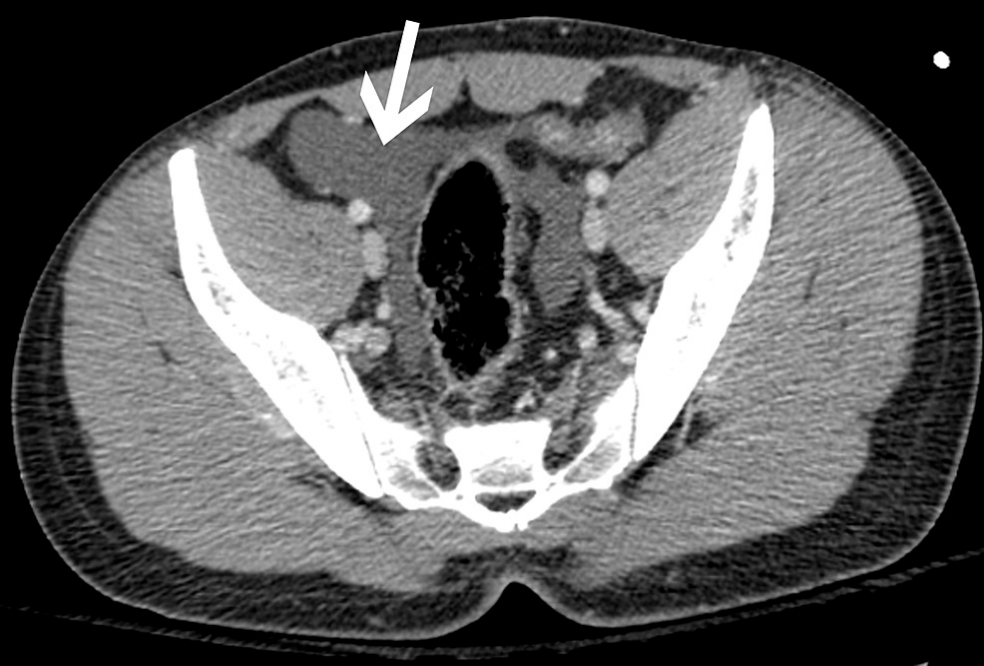
Axial CT image through the pelvis shows ascites (white arrow).

**Figure 3:**
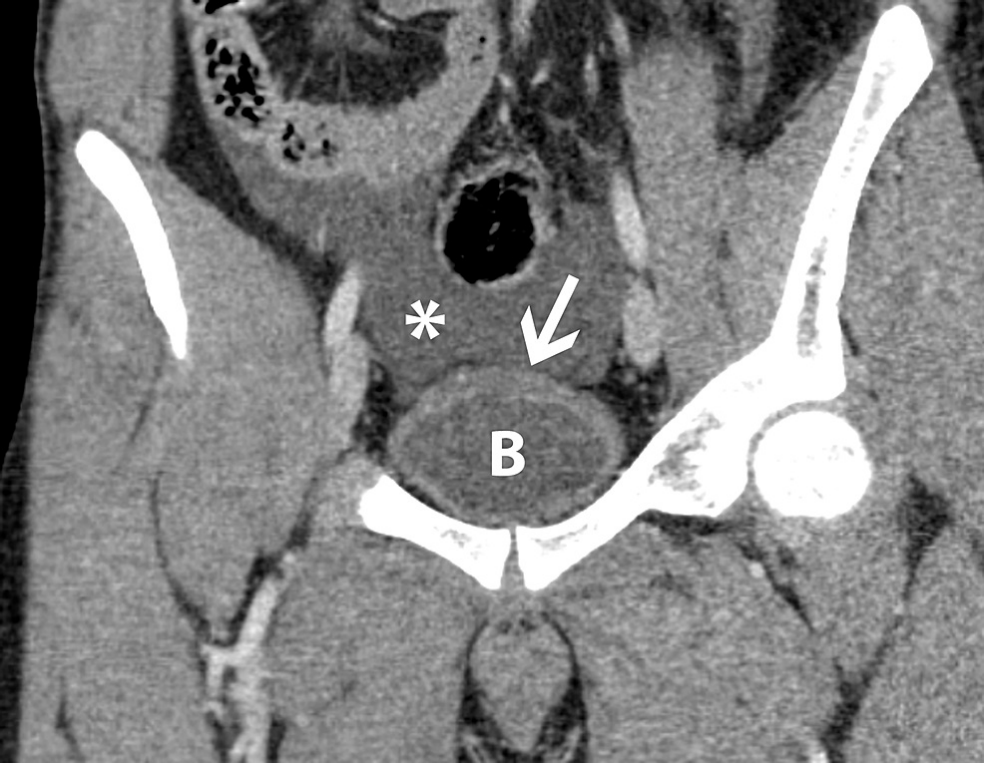
Coronal reformatted CT image shows fluid (white asterisk) in the pelvis superior to the urinary bladder (B). There is mild thickening of the superior bladder surface (white arrow).

A 24-year-old man with no significant medical history presented to our emergency department after being struck by the handlebars of a motorcycle. He complained of constant, severe pain across the lower abdomen. On physical examination, there was diffuse tenderness of the abdomen without visible signs of obvious injury. His vital signs revealed a blood pressure of 142/76 mmHg, heart rate of 93 beats/minute, respiratory rate of 18 breaths/minute, temperature of 36.8°C, and O_2_ saturation (room air) of 99%. Pertinent laboratory values included a slightly elevated creatinine of 1.3 mg/dL and a normal blood urea nitrogen (BUN) of 16 mg/dL. Urinalysis was not performed at presentation, but there was no gross hematuria. Contrast-enhanced CT scan of the abdomen and pelvis showed a moderate amount of simple peritoneal fluid within the upper abdomen and pelvis without evidence of hemoperitoneum or solid organ injury (Figures 1, 2). There was some subtle thickening of the dome of the bladder evident on coronal reformatted views (Figure 3).

**Figure 4:**
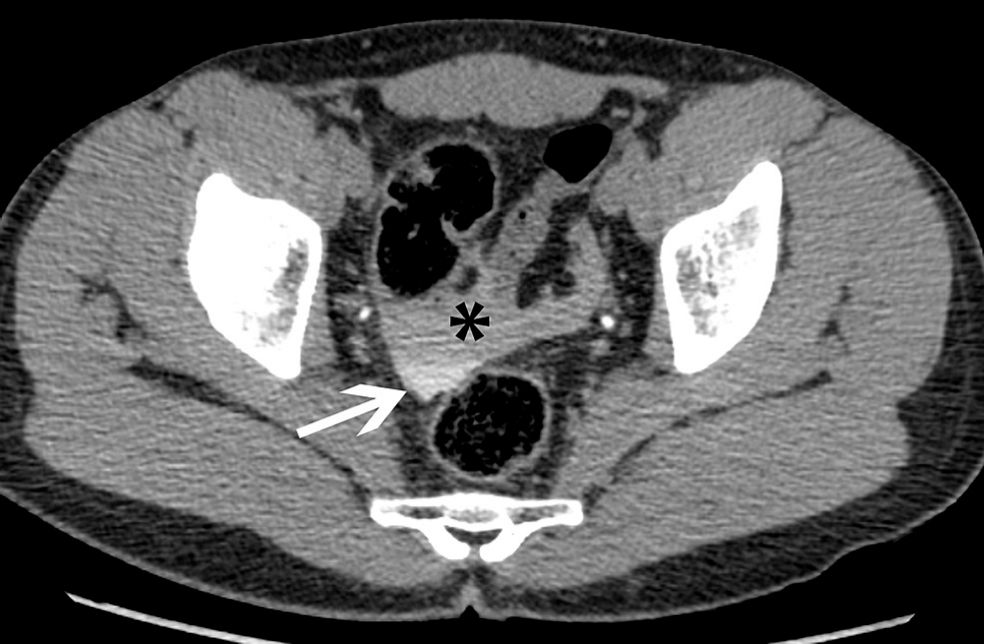
Axial CT image from delayed scan shows high attenuation fluid (black asterisk) in the pelvis representing extravasated contrast material. Note the higher attenuation portion layering dependently (white arrow).

**Figure 5:**
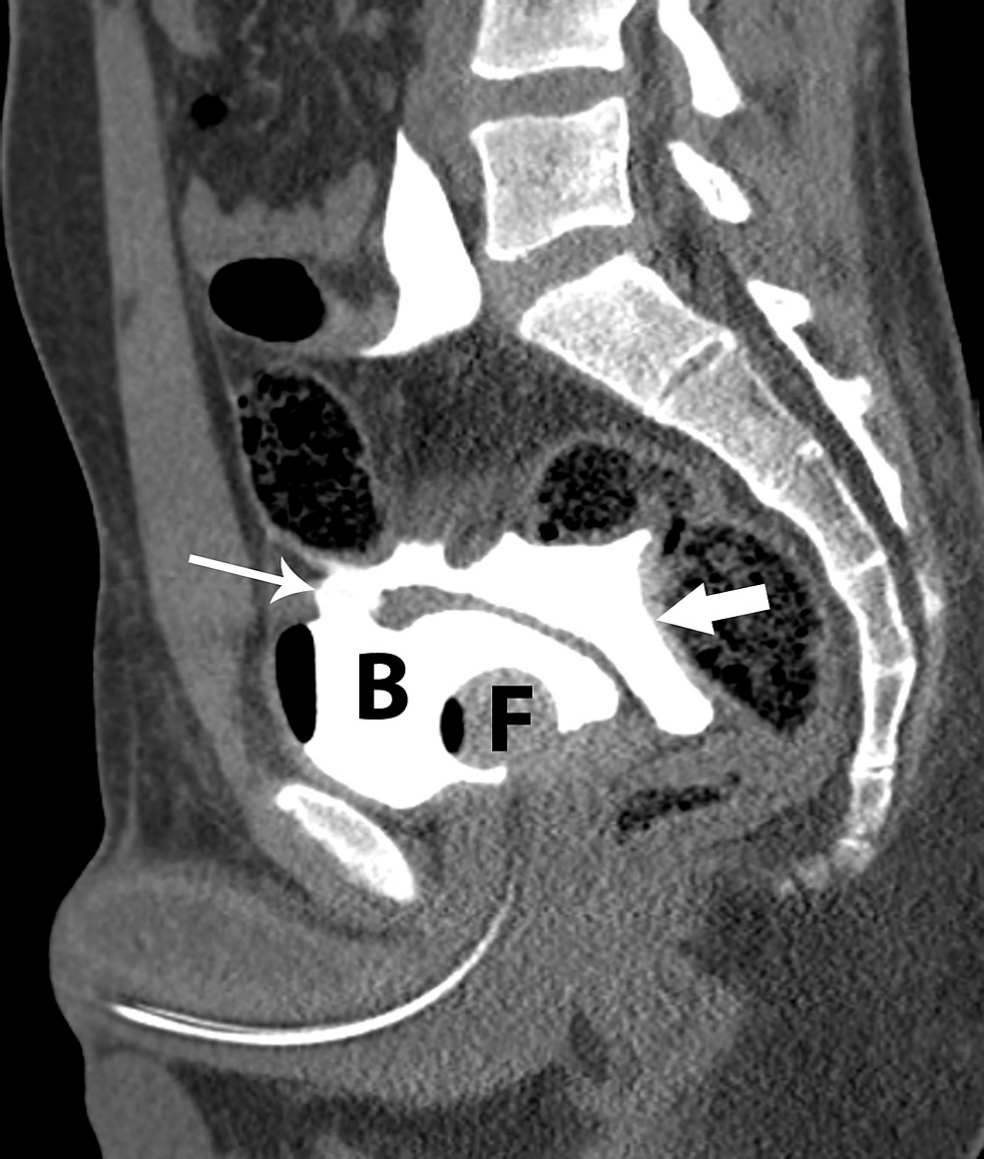
Sagittal reformatted CT image from CT cystogram after injection of contrast material through Foley catheter (F) shows rupture of bladder dome (thin white arrow) with extravasation of dense contrast material (thick white arrow) into the peritoneal space.

**Figure 6:**
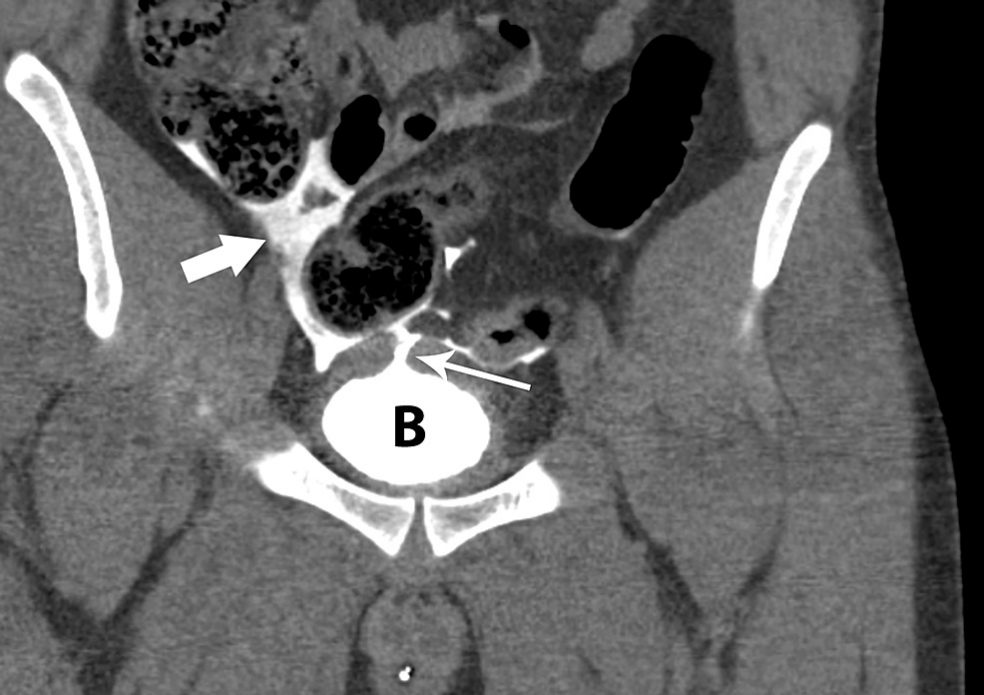
Coronal reformatted image from CT cystogram shows bladder (B) with rupture of bladder dome (thin white arrow) and extravasation of contrast material (thick white arrow) into peritoneal space.

Approximately five hours after the CT scan, the patient complained of urinary retention in the emergency department and a Foley catheter was placed. Hematuria was present upon insertion of the catheter, and the patient was reevaluated with CT of the pelvis due to concern for bladder injury. The repeat CT scan was performed eight hours after the initial CT scan and showed high attenuation fluid in the pelvis representing contrast that had leaked from the bladder into the peritoneal space (Figure 4). CT cystogram was then performed which revealed a tear in the bladder dome with active contrast extravasation into the peritoneal cavity (Figures 5, 6). Vital signs were stable and were not significantly changed from the time of admission. Laparotomy confirmed a 3-cm defect on the bladder dome which was surgically repaired. The patient was discharged three days later, and his recovery was uneventful.

## Discussion

Fluid in the abdominal cavity on CT after blunt trauma is associated with injuries to the spleen, liver, kidneys, small bowel and/or mesentery, bladder, colon and/or rectum, diaphragm, pancreas, and major vessels, in decreasing order of frequency [2]. However, peritoneal fluid without evidence of solid organ injury on CT can be a major diagnostic dilemma, especially in males as it can be associated with small bowel perforation (necessitating laparotomy) or mesenteric injury (which can be managed conservatively in many cases) [2,3]. A small amount of low attenuation fluid in the pelvis may occur without any solid or hollow visceral injury in 3%-5% of male trauma patients [2]. However, if there is a large amount of nonhemorrhagic fluid in the abdomen on CT with no evidence of solid organ injury, after excluding preexisting diseases such as cirrhosis or heart failure, rupture of the urinary bladder should be considered in the differential diagnosis [3,4].

Bladder rupture is rare and occurs in less than 1% of patients with blunt abdominal trauma [1]. Most bladder ruptures are extraperitoneal with intraperitoneal rupture accounting for only 15% [1,5]. The injury often occurs across the bladder dome from direct force to a distended bladder causing an abrupt increase in intravesicular pressure. The bladder dome is mobile and attenuated predisposing it to bulge outward and tear [1,5]. Early recognition of bladder injury is crucial for proper surgical intervention [5]. If intraperitoneal urine is not promptly identified, the toxic metabolites will be resorbed and cause electrolyte derangement, potentially leading to renal failure and sepsis [6]. Mortality can be as high as 20% in these cases [1,6]. If bladder rupture is strongly suspected after blunt trauma, CT cystography is the diagnostic procedure of choice [7,8]. The most typical CT finding of intraperitoneal bladder rupture is extravasation of contrast between loops of small bowel and into the paracolic gutters [5,9]. Intravesical hematoma and heterogeneous attenuation of the bladder dome may also be present [5].

Previous reports describing ascites as the only CT finding of bladder rupture are limited [1,3,9-11]. Our case describes isolated ascites on initial CT with delayed CT and CT cystography confirming bladder rupture. Since modern CT scanners scan the abdomen and pelvis in a matter of seconds after administration of intravenous contrast, imaging of the bladder is usually performed before the phase of contrast excretion into the renal collecting systems and ureters. Unless urinary tract injury is suspected, imaging during the excretory phase is not routinely performed. Thus, in the setting of intraperitoneal bladder rupture, extravasated urine in the peritoneum appears as simple fluid. Our case illustrates that unexplained ascites after blunt abdominal trauma should raise suspicion for bladder injury and prompt careful evaluation of the bladder. The subtle bladder wall thickening along with the dome on the initial CT scan (Figure 3) likely represented a small hematoma. In such cases, delayed CT, after allowing excreted contrast to fill the bladder, can demonstrate the true nature of the abdominal fluid. CT cystography actively distending the bladder with infusion of contrast through a catheter can demonstrate the precise location of the injury.

## Conclusions

Unexplained ascites following blunt abdominal trauma can be a sign of bladder rupture. In such cases, radiologists should have an index of suspicion for bladder rupture, examine the bladder carefully for subtle signs of injury, and consider follow-up delayed CT or CT cystography.
